# Follow-Up by Transcranial Doppler After Rupture of a Giant Intracranial Aneurysm

**DOI:** 10.7759/cureus.31951

**Published:** 2022-11-27

**Authors:** Cláudia I Lemos, Vanessa Almeida, Maria Fátima Soares, Ana Catarina Fonseca

**Affiliations:** 1 Intensive Care Unit, Hospital Central do Funchal, Funchal, PRT; 2 Hemodynamic Laboratory, Department of Neurology, Hospital de Santa Maria, Lisboa, PRT; 3 Department of Neurosciences and Mental Health/Department of Neurology, Hospital de Santa Maria, Faculdade de Medicina de Lisboa, Lisboa, PRT

**Keywords:** external ventricular drainage, giant intracranial aneurysm, elevated intracranial pressure, intracranial vasospasm, transcranial doppler ultrasound

## Abstract

Giant intracranial aneurysms (GIA) are rare and manifest primarily through subarachnoid hemorrhage (SAH), cerebral ischemia, or progressive symptoms of mass effect. Transcranial Doppler (TCD) can be used to monitor cerebral vasospasm after treatment of intracranial aneurysm allowing the adjustment of therapeutics and avoiding complications. The authors present a clinical case of a patient with a ruptured intracranial giant aneurysm in which TCD was essential to monitor vasospasm and intracranial hypertension (IH). A 53-year-old male was admitted due to a sudden headache and impaired consciousness, left hemiparesis, and dysarthria. Cerebral CT scan and CT angiography at admission showed a giant aneurysm of the right middle cerebral artery (MCA) with extensive and diffuse intraventricular SAH of Fisher grade IV and Hunt and Hess grade 4. Clipping, placement of an intracranial pressure sensor, and external ventricular drain (EVD) were performed on the same day, with difficulty in preserving the M2 branch and complicated by postoperative extensive right MCA ischemia. On day three of hospitalization, TCD revealed an increased pulsatility index (>1.5) with clinical deterioration leading to re-intervention for a decompressive craniectomy. On day six, a TCD follow-up was performed to monitor blood flow complications, and particularly vasospasm, showing a severe increase in middle blood flow velocity (MBFV) in the right MCA of 205 cm/s and Lindegaard Index > 6. Daily surveillance by TCD was maintained to guide clinical management since the attempt to withdraw the EVD led to clinical deterioration with subsequent worsening of vasospasm. Improvement occurred after surgery as ventriculoperitoneal shunt insertion was performed. TCD had a major role in the clinical orientation of SAH as well as in intracranial pressure management and was decisive to establish long-term treatment.

## Introduction

With a poorly defined natural history, giant intracranial aneurysms (GIAs; diameter ≥ 25 mm) comprise a minority of all aneurysms (5% incidence) and approximately 5-10% are present in the pediatric population [1]. They tend to be located in regions such as the vertebrobasilar junction, basilar apex, cavernous and supraclinoid carotid, and areas of higher blood flow velocity. GIAs typically present during the 5th and 7th decades and are slightly more common in females [2].

Expected history is unfavorable, as depending on its size (diameter ≥ 30 mm), the rupture risk of intracranial aneurysm commonly intensifies. They carry high mortality (80%) and manifest primarily through subarachnoid hemorrhage (SAH). Symptoms range from gradual neurological deficits to catastrophic SAH and certain patients may suffer multifocal cerebral infarcts or transient ischemic attacks caused by dislodged intra-luminal thrombus [3,4]. In SAH, vasospasm severity correlates with the amount of blood in the subarachnoid space [5].

Transcranial Doppler (TCD) has a major role in the identification and monitoring of vasospasm and intracranial hypertension (IH), therefore, helping in the clinical and therapeutic orientation of SAH. It stands out as the only non-invasive, real-time diagnostic technique that adds hemodynamic information to structural images [6,7].

## Case presentation

**Figure 1:**
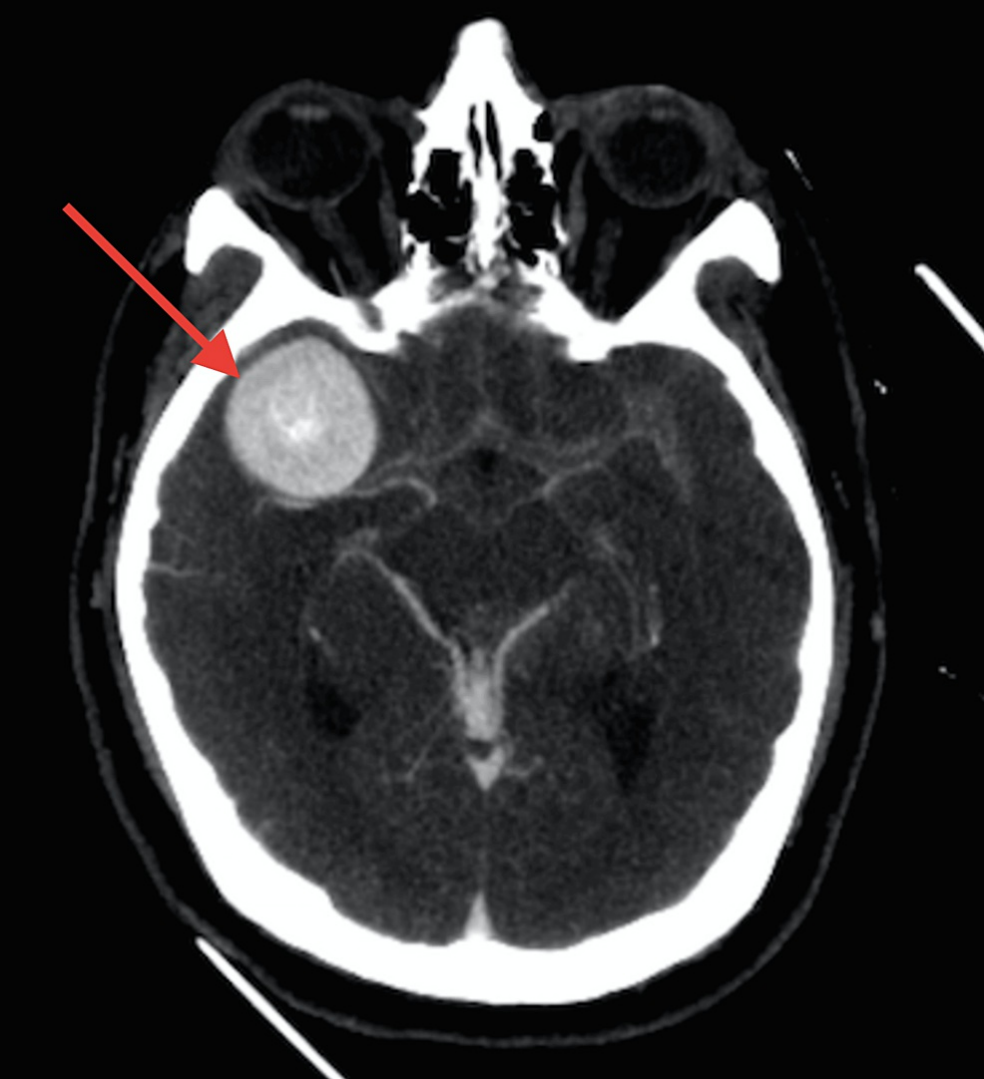
Cerebral CT scan on admission presented a giant aneurysm of the right middle cerebral artery with extensive and diffuse subarachnoid hemorrhage and intraventricular hemorrhage.

**Figure 2:**
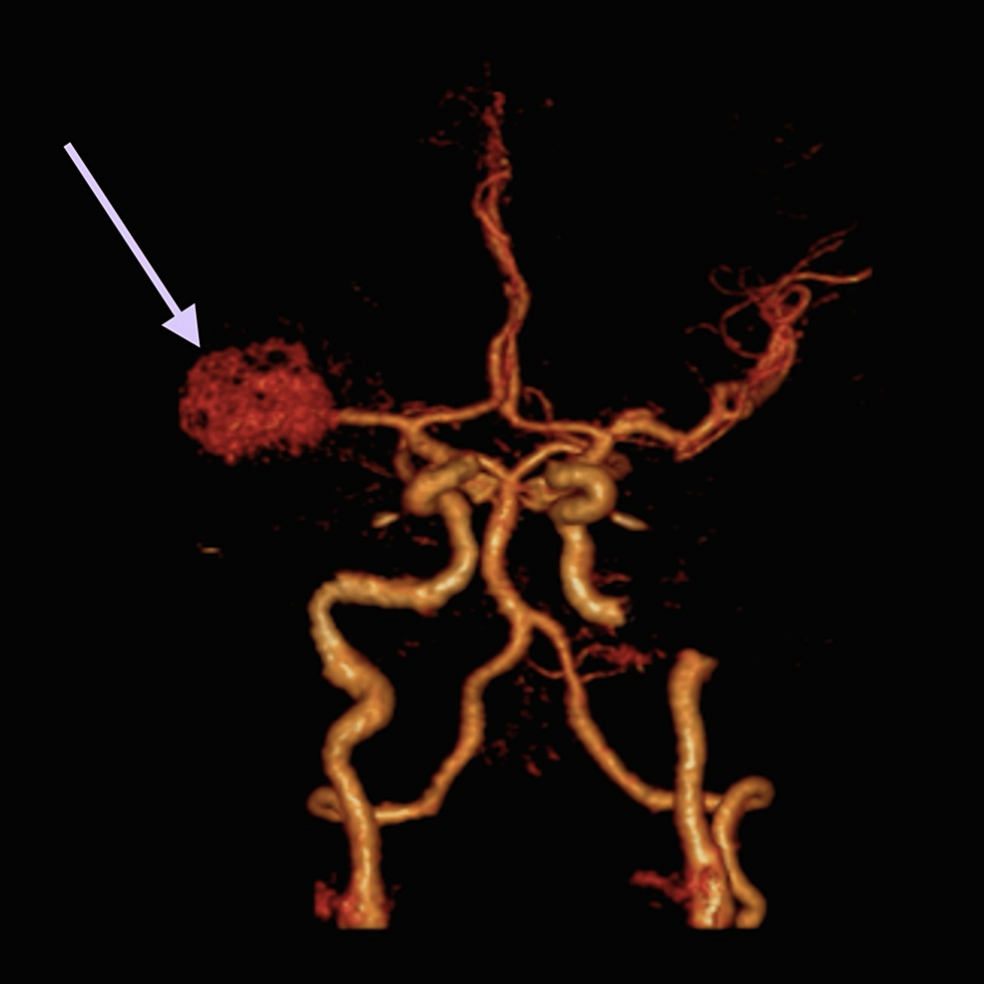
Angio CT on admission identified a giant aneurysm of the right middle cerebral artery.

A 53-year-old male was admitted to the emergency department due to a sudden headache and impaired consciousness. Neurological examination disclosed meningeal signs, left hemiparesis, and dysarthria. Cerebral CT scan and CT angiography displayed an extensive and diffuse intraventricular SAH of Fisher grade IV and Hunt and Hess grade 4 and a giant right middle cerebral artery (MCA) aneurysm measuring 33 millimeters (Figures 1, 2).

Aneurysm clipping and placement of an intracranial pressure sensor and external ventricular drainage (EVD) were performed on the same day. It was impossible to preserve the M2 branch and the procedure was complicated by an extensive right MCA infarct. After the procedure, the patient was admitted to the neurocritical care unit, under invasive mechanical ventilation, and deeply sedated. Vasopressor support with noradrenaline to maintain cerebral perfusion pressure and oral nimodipine were started.

On day three of hospitalization, after suspension of sedation, the patient presented clinical deterioration with an altered state of consciousness and hemiplegia. TCD showed an increased pulsatility index (PI) > 1.5 on TCD evaluation (Table 1) leading to re-intervention for a decompressive craniectomy.

**Table 1:** Relationship between the mean velocities of both anterior and middle cerebral arteries (cm/s), PI value, and EVD placement (cmH20). TCD: transcranial Doppler; SAH: subarachnoid hemorrhage; L MCA: left middle cerebral artery; R MCA: right middle cerebral artery; PI: pulsatility index; EVD: external ventricular drainage.

TCD	Mean velocities, cm/s			
SAH day	L MCA	R MCA	EVD cmH20	PI value
2	55	135	0	<1.3
3	45	125	0	>1.5
4	70	190	0	<1.3
6	125	205	0	<1.3
9	110	160	0	<1.3
10	150	170	15	<1.3
11	130	135	10	<1.3
12	150	155	20	<1.3
13	140	130	10	<1.3
16	145	115	10	<1.3
17	135	135	15	<1.3
18	130	118	10	<1.3
19	105	115	10	<1.3

**Figure 3:**
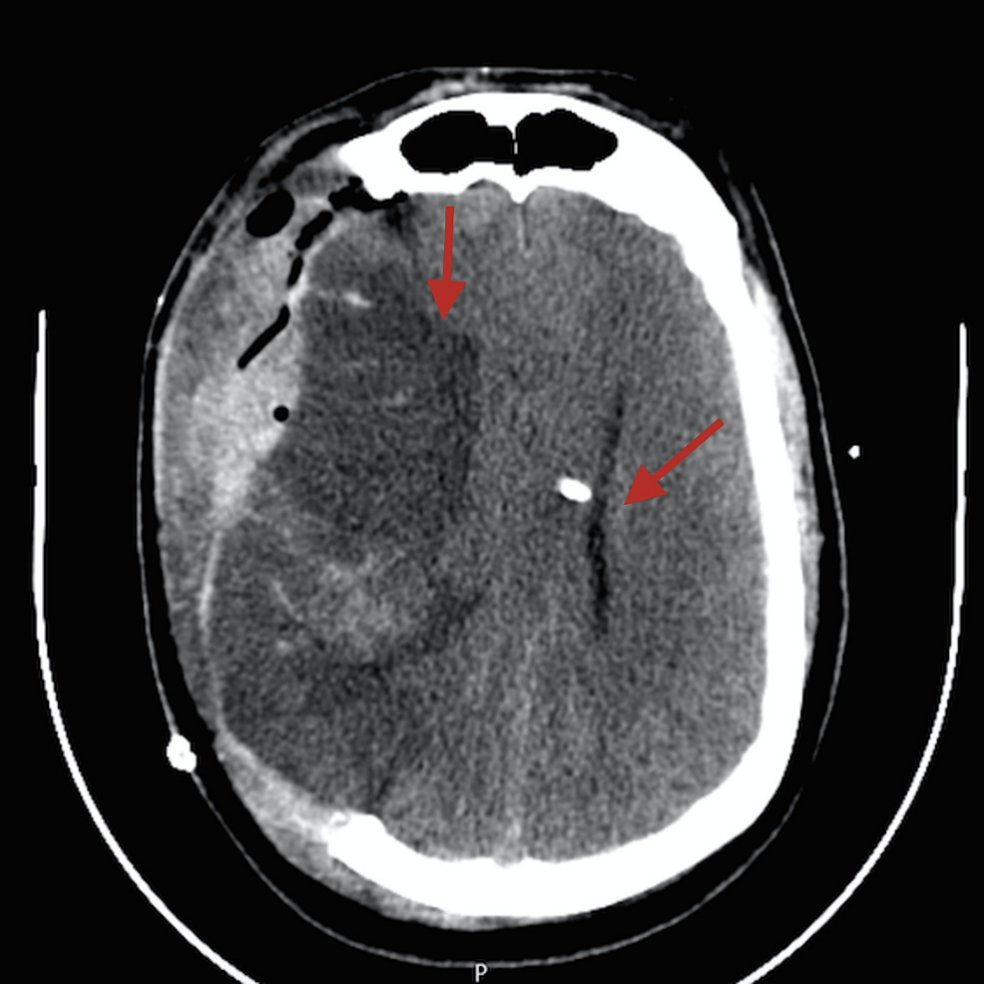
Cerebral CT scan showing ischemic infarction in the territory of the right middle cerebral artery with hemorrhagic transformation, leading to worsening of the mass effect, effacement of the perimesencephalic base cisterns, compression of the ventricular system, and deviation of the midline structures by 11 millimeters.

Postoperative cerebral CT displayed an ischemic infarction in the right MCA with hemorrhagic transformation and mass effect, effacement of the base cisterns, compression of the ventricular system, and deviation of the midline structures (Figure 3).

**Figure 4:**
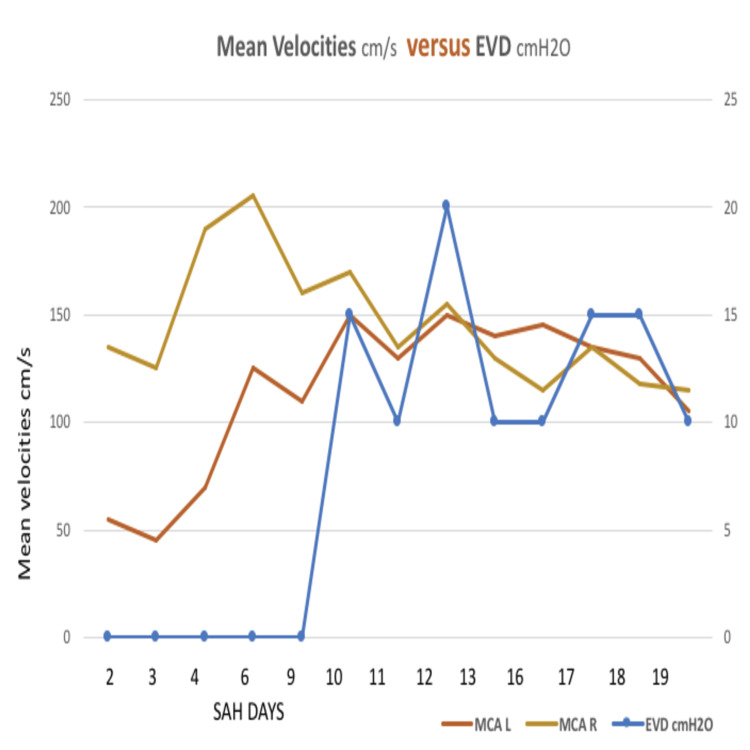
Relationship between the mean velocities of both middle cerebral arteries (cm/s) and EVD placement (cmH20). SAH: subarachnoid hemorrhage; EVD: external ventricular drainage; MCA L: left middle cerebral artery; MCA R: right middle cerebral artery.

On day six, a TCD follow-up was performed to monitor vasospasm. It showed a progressive worsening with mean blood flow velocity (MBFV) of 205 cm/s and Lindegaard Index > 6 in the right MCA. Daily surveillance was maintained for clinical management since the attempt to withdraw the EVD led to clinical deterioration with subsequent worsening of vasospasm (Figure 4). Improvement occurred on day 18 after surgery for ventriculoperitoneal shunt insertion due to chronic hydrocephalus was performed.

## Discussion

In patients with ruptured intracranial aneurysms associated with subarachnoid hemorrhage, timely diagnosis and treatment of cerebral vasospasm (CVS) could be particularly beneficial in reducing morbidity and mortality [8,9]. Also, by insertion of an EVD, the risk of CVS could be reduced since it is a strategy to facilitate the clearance of blood from the subarachnoid spaces because hemolysis of blood is the primary inciting agent for vasospasm [10].

Days after SAH, delayed cerebral ischemia occurs and is part of a post-SAH syndrome. It denotes a treatable cause of morbidity and comprises inflammatory reactions and microthrombosis with arteriolar vasospasm culminating in cortical infarction [11].

For a long time, the most relied exam for the diagnosis of CVS was digital subtraction angiography; however, it is an invasive, relatively expensive, and time-consuming exam and unfeasible to be performed daily [12].

There has been substantial interest in developing noninvasive imaging methods that could detect intracranial aneurysm complications, both in asymptomatic and symptomatic patients. TCD has the advantage compared to digital subtraction angiography of not using ionizing radiation, greater mobility, and lower capital cost; additionally, there are no contraindications and it can be used to monitor cerebral hemodynamics in real time [13,14]. By using the TCD method, it is possible to monitor the efficacy of EVD, which is important to contribute to a better outcome since it can reduce clinical vasospasm and vasospasm-related cerebral infarction in patients with aneurysmal SAH [15].

Klimo et al. [16] suggested that continuous cerebrospinal fluid (CSF) drainage (lumbar or by EVD) has a positive influence in reducing the incidence of CVS and hydrocephalus, improving Glasgow scale score outcomes, and reducing cerebral infarction and mean hospital stay in patients with aneurysmal SAH after surgical clipping. Interestingly, findings in another study by Hoekema et al. [17] indicate that EVD may be inferior to lumbar drainage in subarachnoid clots' washout.

Cerebral ultrasound is an emerging point of care instrument for intensivists, with a significant role in the diagnosis of acute intracranial pathology, such as noninvasive intracranial pressure measurement and of cerebrovascular diseases in the acute clinical setting in the neurocritical unit. Managing TCD, traditionally achieved in the neurosonology laboratory setting, has extended over the last years, opening a new window to the evaluation of cerebral anatomy not only in neurocritical patients but also in general intensive care unit patients [18,19].

PI provides information regarding quantitative alterations in the morphology of the TCD waveform. It is inversely proportional to the mean cerebral perfusion pressure, so, it increases with IH. In our particular case, the patient suffered clinical deterioration and presented an increased PI > 1.5 in TCD, suggesting IH. This information corroborated the need for surgical re-intervention [20].

The spectral waveform derived from TCD is characterized by three components: cerebral flow peak systolic velocity, end-diastolic velocity, and mean flow velocity. However, an increase in velocity alone is not sufficient to reach a vasospasm diagnosis, given that several situations may cause velocities to increase, for example, in cases of stenosis, anemia, hypervolemia, or hyperthermia. Subsequently, the Lindegaard Index was introduced to increase the sensitivity of vasospasm diagnosis [20]. Our case reports a gradual worsening with the highest MBFV of 205 cm/s in the right MCA and a Lindegaard Index > 6, which indicated a severe vasospasm that determined emergent treatment.

In this case, daily surveillance with TCD was maintained to guide the clinical and therapeutic management of the patient, since the attempts to withdraw the EVD led to clinical deterioration with subsequent worsening of vasospasm, without compromising PI. This case culminated with ventriculoperitoneal shunt insertion due to chronic hydrocephalus after 18 days. Professional healthcare workers should be aware of the role that TCD may have in the surveillance of vasospasm and therefore prevention of delayed ischemia. The severity of vasospasm may correlate with CSF drainage.

## Conclusions

TCD was vital for therapeutic decisions. Permitting real-time information and early diagnosis of the patient’s clinical oscillations related to variations in EVD played a key role in the anticipation of complications derived from prolonged vasospasm. These conclusions support recent studies where CSF drainage may relate to the duration and severity of vasospasm.
